# Supplementary material for: A high-resolution mRNA expression time course of embryonic development in zebrafish
Source: eLife. 2017 Nov 16;6:e30860. doi: 10.7554/eLife.30860 (PMC5690287; doi:10.7554/eLife.30860)
Supplement: Supplementary file 6. [file elife-30860-supp6.zip › biolayout-clusters-files/Cluster145-genes.html]

Cluster145


# Cluster145: Genes

| Ensembl ID | Gene Name | Chr | Start | End | Biotype |
| --- | --- | --- | --- | --- | --- |
| ENSDARG00000024030 | angptl2a | 5 | 4372659 | 4404631 | protein\_coding |
| ENSDARG00000042934 | ctgfa | 20 | 25441724 | 25444400 | protein\_coding |
| ENSDARG00000099371 | cygb1 | 3 | 6753948 | 6783800 | protein\_coding |
| ENSDARG00000078359 | endouc | 18 | 15490813 | 15498934 | protein\_coding |
| ENSDARG00000061665 | hcn2b | 22 | 18927803 | 18982990 | protein\_coding |
| ENSDARG00000057737 | rxraa | 21 | 17731440 | 17910993 | protein\_coding |
| ENSDARG00000017369 | sema3d | 18 | 9213759 | 9280656 | protein\_coding |
| ENSDARG00000071585 | si:dkeyp-110e4.11 | 14 | 26248070 | 26264126 | protein\_coding |
| ENSDARG00000020072 | thbs4b | 21 | 4964948 | 4998122 | protein\_coding |
